# Antibiotic Resistance in *Enterococcus faecalis* Isolated from Hospitalized Patients

**DOI:** 10.5681/joddd.2013.018

**Published:** 2013-05-30

**Authors:** Esrafil Balaei Gajan, Adileh Shirmohammadi, Mohammad Aghazadeh, Mohammad Alizadeh, Alireza Sighari Deljavan, Farzin Ahmadpour

**Affiliations:** ^1^Dental and Periodontal Research Center, Tabriz University of Medical Sciences, Tabriz, Iran; ^2^General Dentist, Department of Community Dentistry, Faculty of Dentistry, Tabriz University of Medical Sciences, Tabriz, Iran; ^3^Associate Professor, Department of Periodontics, Faculty of Dentistry, Tabriz University of Medical Sciences, Tabriz, Iran; ^4^Assistant Professor, Department of Microbiology, Faculty of Medicine, Tabriz University of Medical Sciences, Tabriz, Iran; ^5^Lecture, Faculty of Nursery and Midwifery, Tabriz University of Medical Sciences, Tabriz, Iran; ^6^Reaserch Assistant, Faculty of Dentistry, Tabriz University of Medical Sciences, Tabriz, Iran; ^7^Dental Student, Faculty of Dentistry, Tabriz University of Medical Sciences, Tabriz, Iran

**Keywords:** Antibiogram, Enterococcus faecalis, MIC

## Abstract

**Background and aims:**

Enterococci are Gram-positive cocci that often occur in pairs (diplococci) or short chains. Be-side developing high level of antibiotic resistance, these bacteria can cause wide range of disease in human, thus to help provide an effective treatment for infections caused by this genus, this study was conceived to provide information on Enterococcus faecalis Antibiotic resistance to widely used antibiotics in hospitalized patients.

**Materials and methods:**

Disk diffusion agar and Broth dilution methods were used to perform Antibiogram test on isolated Enterococcus faecalis. Culture medium used for Disk diffusion agar test was Muller Hinton agar, and for Broth dilution methods, Muller Hinton broth culture medium was utilized. In disk diffusion agar method, different commercial antibiotics disks produced by Pharmaceutical companies were used. Microsoft Excel software was used to perform statistical analysis.

**Results:**

Based on antibiograms of 105 cases, a high resistance to Synercid, Nalidixic acid, Oxacillin and Teofilin was de-tected whereas the lowest resistance observed in Nitrofurantoin, Vancomycin, Linezolid and Teicoplanin antibiotics.

**Conclusion:**

According to the results, Teicoplanin, Vancomycin, Linezolid and Nitrofurantoin are recommended against E. faecalis species.

## Introduction

Enterococci are Gram-positive cocci that commensal inhabitants of the human intestine. Some strains of this genus have developed resistance to antibiotics.^1^ These bacteria are non-motile, without capsule and cultivated on the bile esculin agar (BEA) as a selective differential medium. Enterococcus growths fast in 37-42°C temperature and forms non-hemolytic colonies.^2^ Members of the genus Enterococcus are capable of growing in the presence of 6.5% NaCl and 4% bile (oxgall) and hydrolyzing esculin to glucose and esculetin. Esculetin combines with ferric ions to produce a black complex.^2^ Important clinical infections caused by Enterococcus include urinary tract infections, bacteremia, bacterial endocarditis, diverticulitis, and meningitis. They can tolerate 60°C heat for 30 minutes.^3-5^

This study was conceived to provide information on Enterococcus faecalis Antibiotic resistance to widely used antibiotics in hospitalized patients, considering dose and their type.

## Materials and Methods

One-hundred and five samples were collected from patients hospitalized with enterococcus infections in Tabriz, Iran, from March 2012 to the end of June 2012.

The most simple and best methods for antibiogram are preparation tubular tenuityies and disk diffusion agar. Bacterial suspensions with McFarland Standard 0.5% were cultured in Mueller-Hinton agar medium and impregnated paper disks were placed on the culture medium.^2,3^0.5% McFarland standard was prepared by mixing 0.05 ml of 1.175% barium chloride dihydrate (BaCl_2_•2H_2_O), with 9.95 ml of 1% sulfuric acid (H_2_SO_4_). The mixture was confirmed by spectrophotometer at 625 nm wave length. The absorbency for 0.5% McFarland standard must be in 0.1-0.68 range. The standards are equivalent to 1-1.5×10^8^ bacteria in milliliter.

The antibiotic disks used for antibiogram test included Vancomycin (30 mg), Nalidixic Acid (30 mg), Ampicillin (10 mg), Nitrofurantoin (300 mg), Oxacillin (1mg), Tetracycline (30 mg), Gentamicin (100 mg), Co-trimoxazole (1.25-22.75), Erythromycin (30 mg), Teicoplanin (30mg), Linezolid (30 mg), Ciprofloxacin, Synercid, Doxycycline.

After 24 hours of incubation in 37°C, the inhibition zone was measured with metric ruler and then matched with NCCLS (National Committee for Clinical Laboratory Standards) tables to finalize antimicrobial susceptibility tests.

Minimum inhibitory concentration (MIC) was used to determine antimicrobial resistance.

**Table 1:** Percentage of resistant enterococci to different antibiotics

Antibiotics	Synercid	Nalidixic acid	Oxacillin	Teicoplanin	Ciprofloxacin	Doxycycline	Ampicillin	Gentamycin	Erythromycin	Amikacin	chloramphenicol	Nitrofurantoin	Linezolid	Vancomycin	Teicoplanin
Resistant (%)	100	94	93	83	44	44	4	36.2	29	27	2	18.6	5.1	3.6	3.3

Antibiotic with different concentrations were prepared from antibiotic solution. ^1,6,7^12 sterile tubes in the form of tower row triplet and added 1, 3 and 7 ml Muller Hinton broth culture environment to first, second, and third rows, respectively. Then, we added 1 ml of antibiotic solution to three first rows. 3 ml was taken from the third tube and added 1 ml to second row of triplet tube. After mixing, 3 ml was taken from sixth tube again, and 1 ml was added to the third row of triplet tubes. Again 3 ml was taken from ninth tube and 1 ml was added to the fourth row of triplet tubes. Accordingly, antibiotic concentrations were prepared in tubes 1 to 12 as 1024, 512, 256, 128, 64, 32, 16, 8, 4, 2, 1, 0.5, and 0.25, respectively. 1 ml of each concentrations was poured in the 12 sterile tubes and then bacterium suspensions were added (0.5 McFarland 100 land and 19.9 ml Muller Hinton broth environment). There are 1.5×10^8^ bacteria in each milliliter, and 1 ml was added to each of the 12 tubes. Then, the tubes were placed in incubator at 37°C for 24 hours.^6,8^ The development of opacity in the tubes indicated bacterial resistance. MIC is the case antibiotic.^8,9^To determine resistance percentage and distinguish multi-resistance lineage of Enterococcus faecalis isolated from patients Microsoft Excel software was used.

## Results

Based on antibiograms of 105 subjects, the presence of antibiotic-resistance was more prevalently observed with Tetracycline, Oxacillin and Nalidixic Acid. Also, a 100% resistance was observed in the case of Synercid. Percentage of enterococcus antibiotic-resistance to different antibiotics is shown in the Table 1.

## Discussion

For effective treatment of bacterial infections, adequate concentration of antibiotic must be present in the site of infection. This concentration must show either bactericidal or bacteriostatic activity against susceptible bacteria. Therefore, if in a case, the level of antibiotic required for preventing bacteria from growing, exceeds toxic concentrations, the bacterium is considered antibiotic-resistant.

Enterococcus faecalis is Gram-positive cocci that often in the form of diplococci or short chains. E. faecalis can cause endocarditis and bacteremia, urinary tract infections (UTI), meningitis, and other infections in humans. This species can grow in bile esculin agar and environments with 6.5% NaCl. It growths fast at 37-42°C temperature while it can tolerate 60°C heat for 30 minutes.

Recent reports show that resistance of these bacteria to commonly used antibiotics is increasing worldwide. Even Vancomycin-resistant and Gentamicin-resistant species of E. faecalis are reported.^10,11^ An antibiogram test is, hence, required for effective treatment. In the present study, the highest rate of resistance was seen with Synercid (100%). For Nalidixic acid, resistant was 94% and for Oxacillin it was about 93%. Also about 83% of cases showed resistance to Teofilin which is regarded a high resistance. These antibiotics cannot be appropriate prescriptions for the treatment of E. faecalis infections. Moderate resistance was reported in Ciprofloxacin, Doxycycline, Amikacin, Gentamycin, and the least percentage of resistance was obtained with Teicoplanin, Vancomycin, Linezolid, and Nitrofurantoin.

According to the results, it can be concluded that instead of prescribing antibiotics like Gentamycin, Amikacin or Oxacillin against E. faecalis infection, antibiotics like Teicoplanin, Vancomycin, Linezolid, and Nitrofurantoin are recommended along with antibiotics like chloramphenicol and Ampicillin as the second-line agents.
